# Clinical relevance of esophageal subepithelial activity in eosinophilic esophagitis

**DOI:** 10.1007/s00535-019-01624-3

**Published:** 2019-09-12

**Authors:** Ikuo Hirano

**Affiliations:** Division of Gastroenterology and Hepatology, Northwestern University Feinberg School of Medicine, 676 North Saint Clair, Suite 1400, Chicago, IL 60611 USA

**Keywords:** Eosinophilic esophagitis, Dysphagia, Eosinophilic gastrointestinal disease, Esophageal stricture

## Abstract

Esophageal subepithelial activity (ESEA) is an important determinant of disease severity and complications in eosinophilic esophagitis (EoE). Inflammation and fibrosis of the lamina propria and muscularis propria result in esophageal dysfunction and stricture formation that are clinically manifest by symptoms of dysphagia and food impaction as well as the need for esophageal dilation. Esophageal biopsies that are limited to the evaluation of the esophageal epithelium are an inadequate means to assess overall, clinical disease severity in EoE. Instruments for the assessment of subepithelial activity in EoE are both limited and/or underutilized and thus represent an important unmet clinical need. Studies using endoscopic features, endoscopic ultrasonography, and barium esophagography have demonstrated improvement in ESEA parameters with topical steroid therapy. Impedance planimetry is being evaluated as an objective and quantifiable measure of esophageal distensibility that is a consequence of ESEA. In conjunction with symptom and histologic assessment, evaluation of ESEA provides a more complete evaluation of disease activity in EoE that will enhance clinical care as well as provide insights into the strengths and limitations of therapeutic interventions.

## Introduction

Eosinophilic esophagitis (EoE) is a chronic, immune/antigen-mediated esophageal disease characterized by symptoms related to esophageal dysfunction and eosinophil-predominant esophageal inflammation. In both clinical practice and clinical trials, disease activity assessment in EoE centers upon symptoms and histopathology. Symptoms as well as disease complications of stricture formation and food impaction have been more closely associated with esophageal subepithelial activity (ESEA) than esophageal mucosal eosinophilic inflammation [1-5]. This concept is further evidenced by the immediate relief of dysphagia that occurs in response to esophageal dilation in the absence of improvement in esophageal mucosal inflammation [6]. ESEA includes lamina propria fibrosis, subepithelial inflammatory infiltration, esophageal dysmotility, and esophageal stricture formation. Esophageal biopsies that sample the esophageal epithelium, therefore, are an inadequate means to assess overall disease severity in EoE.

The purpose of this review is to summarize current data regarding the clinical assessment of ESEA in EoE. Such evaluation provides a more complete depiction of disease activity in EoE that enhances clinical care as well as provides insights into the strengths and limitations of therapeutic interventions.

## Clinical methods to assess esophageal subepithelial disease activity in EoE

### Esophageal biopsy sampling

**Fig. 1:**
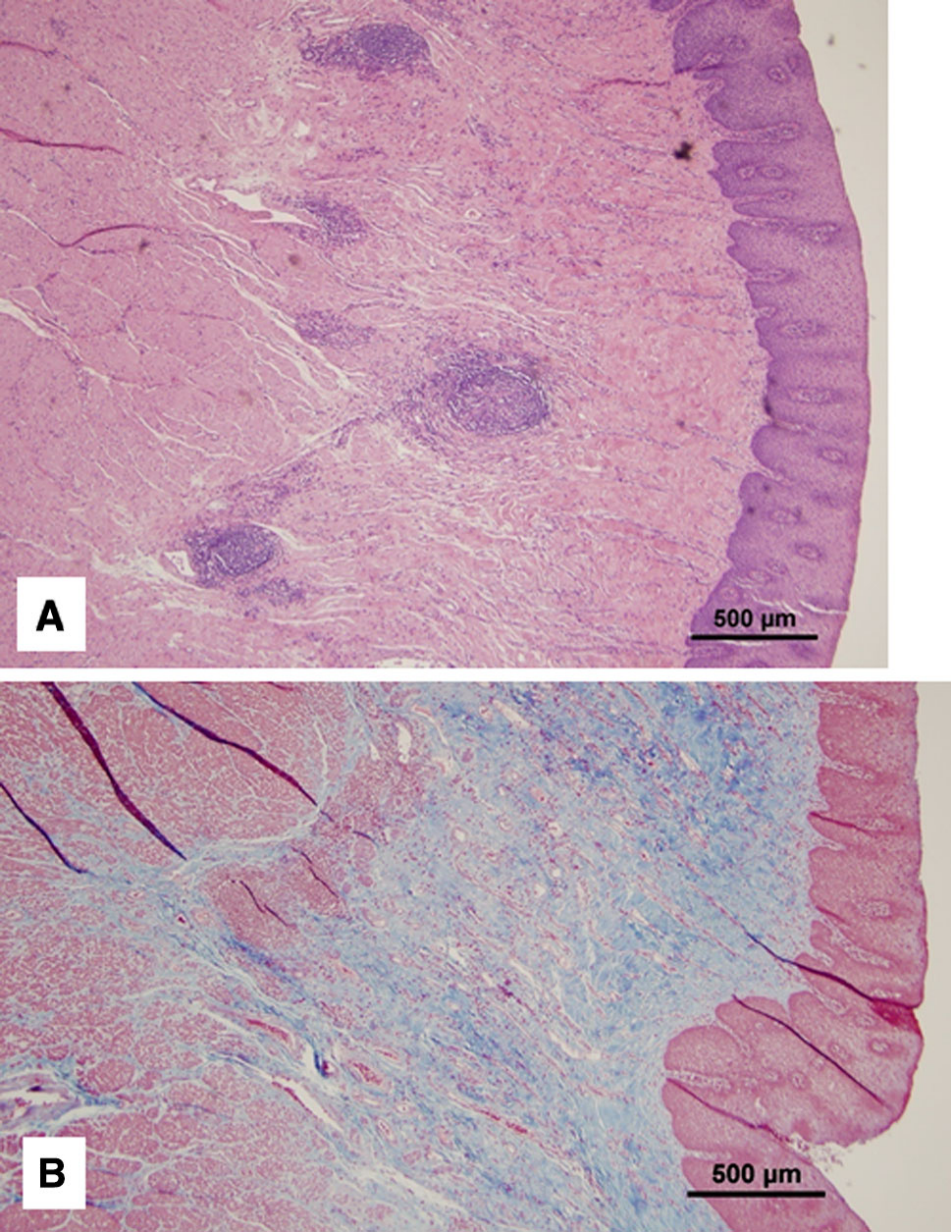
Full-thickness esophageal histology in EoE demonstrates eosinophilic inflammation in the mucosa, submucosa and muscularis propria (**a**). Trichrome staining of the same specimen demonstrated transmural esophageal fibrosis

Standard esophageal biopsies that sample the esophageal epithelium are intrinsic to the diagnosis of EoE. In a prospective placebo-controlled, clinical trial of budesonide oral suspension, lamina propria deemed adequate for histologic evaluation by a centralized, expert pathologist was only found in 37% of esophageal biopsies [7]. A retrospective report of 30 adult patients compared the yield of subepithelial tissue using various biopsy forceps [8]. Large capacity forceps demonstrated subepithelial sampling in 55% compared to 90% for a specialized, “static jaw’” type forceps. The high yield of the asymmetric forceps’ design was confirmed in a second retrospective study of 200 adults where subepithelial tissue was evaluable in 87% [9]. In this same study, esophageal epithelial eosinophil density only modestly correlated with subepithelial eosinophilic inflammation (rho 0.33). In one-third of patients, eosinophil density was greater in the subepithelium than the epithelium. Furthermore, subepithelial fibrosis was demonstrated in 82%. Evaluation of cases of full thickness esophageal histology in EoE has demonstrated eosinophilic inflammation, eosinophil-related mediators, and fibrosis extending not only into the submucosa but also muscularis propria (Fig. 1) [10].

A muscular variant of EoE has been described in association with hypercontractile esophageal motility disorders that include jackhammer esophagus, achalasia and distal esophageal spasm. Deeper esophageal biopsies by means of tunnel biopsies or cap-assisted, endoscopic mucosal resection methods sampling the muscularis mucosa or muscularis propria demonstrated smooth muscle infiltration by eosinophils [11, 12]. Sato and colleagues reported a case series of four patients with hypercontractile esophageal dysmotility (three with jackhammer and one with nutcracker esophagus) that were diagnosed at the time of a per oral esophageal myotomy [13]. Interestingly, none of the patients had eosinophils in the epithelium. This group also reported a patient with normal epithelial but elevated subepithelial eosinophilia. These cases are similar to early reports of four patients with a muscular variant of EoE, three of whom were noted to have normal esophageal mucosal biopsies but marked eosinophilic infiltration of the muscularis propria based on tissue obtained at the time of surgery or endoscopic mucosal resection in one case [11, 12, 14].

In summary, subepithelial esophageal tissue sampling methods are providing important insights into the role of lamina propria and muscularis propria inflammation and fibrosis in EoE. The data supports the concept of transmural pathologic alterations in eosinophilic esophagitis that may account for the dissociation between clinical outcomes and mucosal pathology readouts.

### Radiologic imaging

**Fig. 2:**
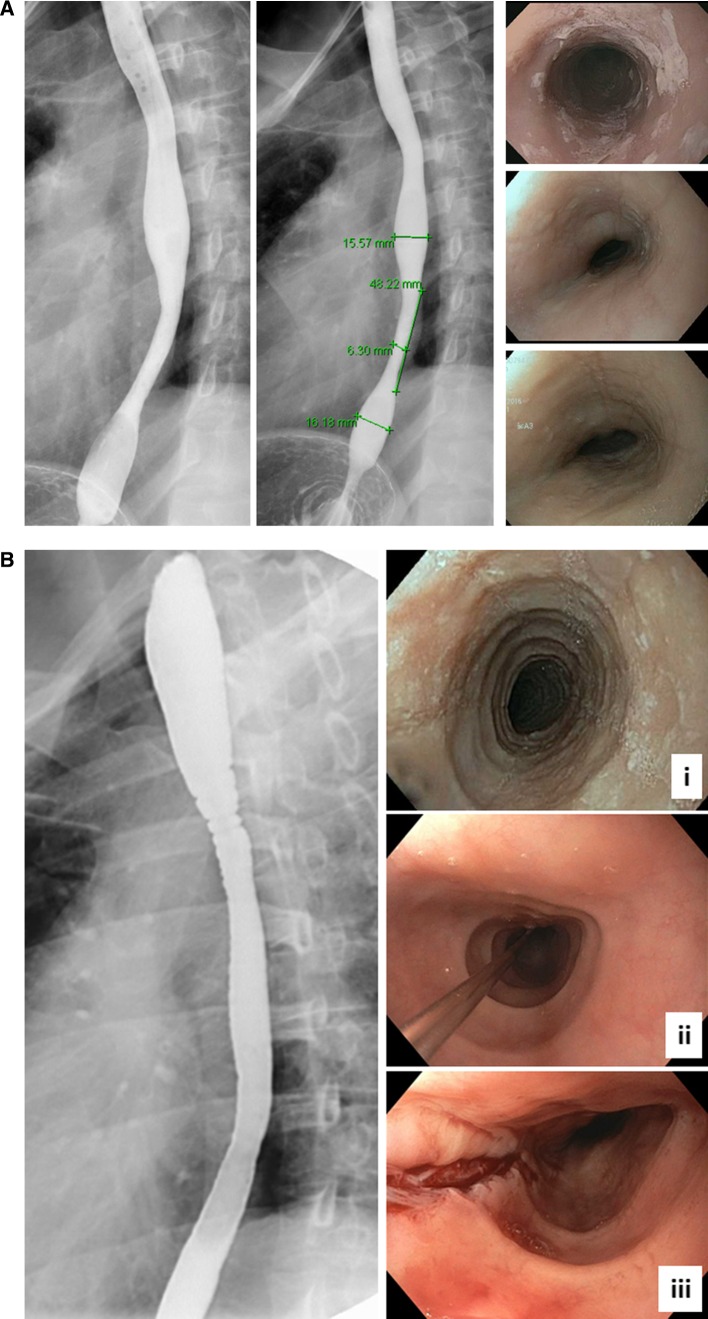
Narrow caliber esophagus in EoE can be a smooth diffuse tapered appearance (**a**) or a more typical “trachealization” with distinct ring-like deformation (**b**). Inflammatory features are more apparent on endoscopic imaging compared to barium esophagram (**a**). With medical therapy of EoE, inflammatory features may resolve but remodeling changes can persist (**b**, **i** pretherapy, **b**, **ii** post topical steroids). Such patients often require esophageal dilation to alleviate persistent dysphagia related to stricture formation (**b**, **iii**)

Barium radiography represents a traditional method to evaluate esophageal structure and function. Early case series described the association of marked restriction of the esophageal luminal caliber with EoE, characterized as a narrow caliber or small caliber esophagus (Fig. 2a) [15]. Additional early case series noted multiple, ring-like stenoses spanning lengths of the esophagus were initially confused with congenital esophageal stenosis but were subsequently recognized to be a characteristic feature of EoE (Fig. 2b) [16]. Alexander characterized restriction of the esophageal diameter in a cohort of adults with EoE, demonstrating a reduction in esophageal luminal diameters compared with controls [17]. Studies have demonstrated the substantial inaccuracy of endoscopists’ assessment of esophageal stricture in pediatric and adult EoE when compared with radiologic assessment [18, 19]. In the adult study, endoscopy had a sensitivity of only 25% for esophageal strictures of less than 16 mm and 33% for strictures less than 14 mm [18]. A second, recent study comparing endoscopic with radiologic findings in adults with EoE noted greater detection of strictures but significantly lower detection of edema, furrows and exudate with barium radiography (Fig. 2a, b) [20]. Of note, narrow caliber esophagus in EoE can be arbitrarily defined as narrowing less than 18 mm involving greater than 50% of the esophageal length [21].

Radiologic assessment of ESEA is widely available but is limited by the inability to control for intraluminal distension pressure. A small volume of barium with low intrabolus distension pressure will tend to provide falsely low estimates of the diameter of an esophageal stricture since the stiffness of the esophageal wall limits the ability of the wall to expand. Limited studies have used cross-sectional imaging modalities such as computed tomography (CT) or magnetic resonance imaging (MRI) to characterize the intramural effects of EoE [11, 22].

### Endoscopy

Endoscopically detected esophageal features of EoE include longitudinal furrows, white exudates (plaques), edema (loss of vascular markings), rings (trachealization), and strictures (Fig. 2a, b). Prospective studies in EoE have identified endoscopic abnormalities in over 90% of children and adults with EoE [23, 24]. Specific endoscopic features in patients with EoE have been shown to vary by age. Younger patients are more likely to have findings of exudates, furrows, edema whereas adult patients are more likely to have strictures, rings, narrow caliber esophagus, and crepe-paper mucosa [24, 25]. Strictures and lumen compromising rings that are manifestations of ESEA are commonly identified in adults with EoE but a minority of pediatric EoE patients. Esophageal strictures are uncommonly identified in children (< 5% of EoE subjects), even though food impactions occur in up to 30% of subjects [25]. In adults, strictures defined as a reduction in luminal diameter to less than 10 mm have been reported in as many as 38% [26]. As discussed below, studies using barium radiography or impedance planimetry show a significantly greater prevalence of esophageal strictures than those using endoscopy.

Duration of the untreated disease has been associated with increased risk of endoscopically identified esophageal stricture, supporting the concept of progressive esophageal ESEA in EoE that may explain phenotypic differences between children and adults [2, 26, 27]. The endoscopic findings correlate with typical clinical presentations that are characterized by anorexia/early satiety, GERD-like symptoms and dysphagia in children and dysphagia with food impaction in adults [3, 28]. These observations support an important distinction in the prevalence of ESEA consequences of esophageal eosinophilia in different age groups and the concept of progressive esophageal stricture with increasing duration of disease.

**Fig. 3:**
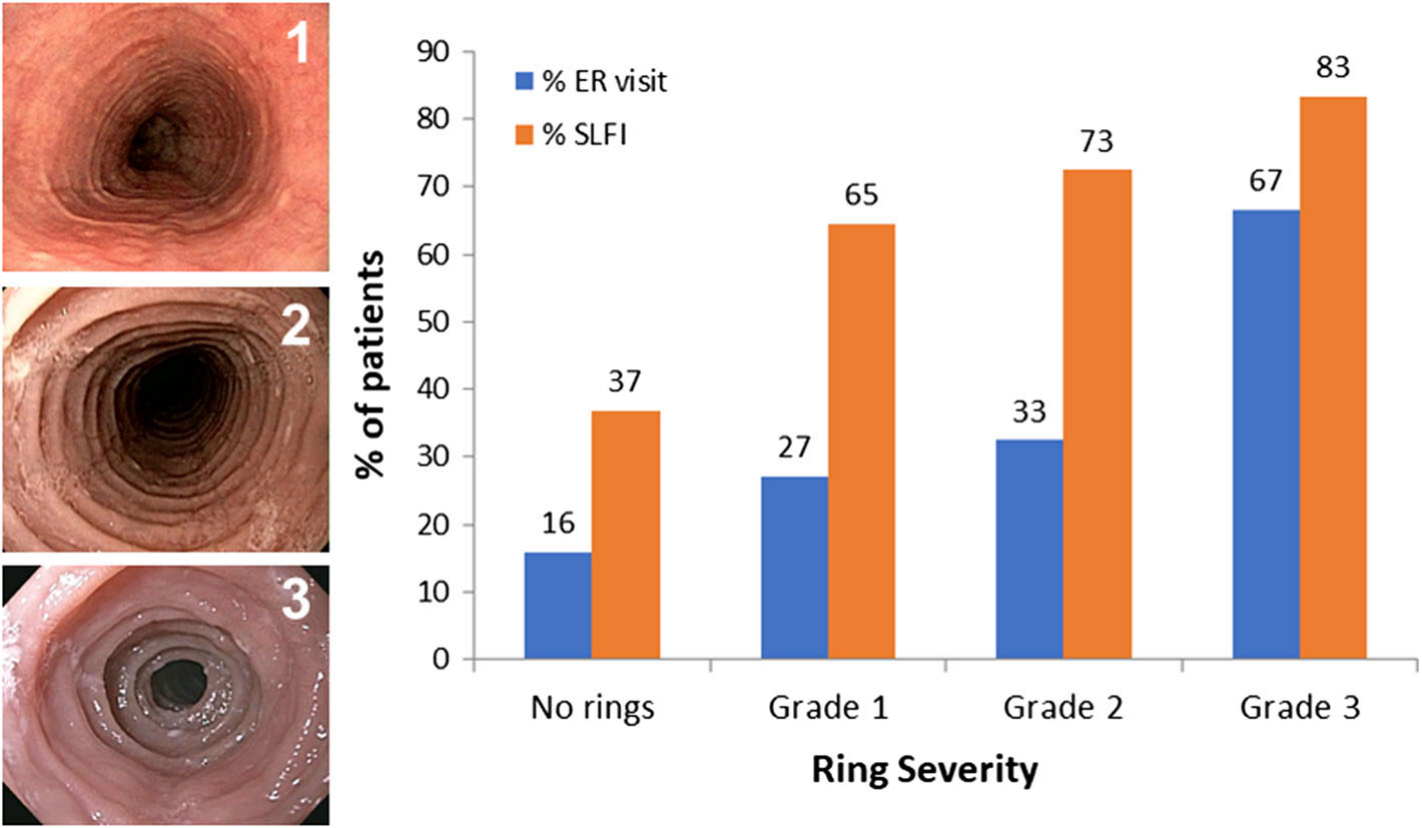
(Left) Esophageal ring severity can be graded as mild (grade 1), moderate (grade 2), or severe (grade 3) based on endoscopic imaging. (Right) Both self-limited food impaction (SLFI) and emergency room visits for food impaction (% ER visit) increase with higher degrees of severity of esophageal rings as demonstrated on endoscopy [2]

A classification and grading system to assess the endoscopic findings in EoE has been developed and validated in terms of inter and intra-observer agreement as well as responsiveness to medical and dietary intervention [29]. The acronym for the Endoscopic REFerence System, EREFS, designates the five major features of EoE (Edema, Rings, Exudates, Furrows, Stricture). This instrument was created to standardize and grade the endoscopic assessment performed by gastroenterologists. EREFS is an important tool that accounts for aspects of subepithelial esophageal activity that are not currently captured in routine pathology reports. To emphasize the significance of endoscopically detected remodeling, the occurrence of food impaction, a clinically relevant symptom outcome of EoE, has been shown to be associated with the assessment of ring severity using the EREFS system (Fig. 3) [2, 3, 30].

In summary, identification and grading of endoscopically identified esophageal features of EoE detects both inflammatory and remodeling aspects of disease activity in EoE. Endoscopic outcomes complement the current use of symptoms and mucosal pathology as the primary outcome metrics in clinical trials of novel therapies in EoE. Further validation of scoring metrics for endoscopic features that optimize the responsiveness to therapy will enhance their utilization as a therapeutic endpoint, paralleling the increasing importance of endoscopic outcomes in inflammatory bowel disease.

### Endoluminal ultrasonography

Endoscopic ultrasonographic demonstration of the expansion of the muscularis propria was described in an early case report of an elderly patient with a muscular variant of eosinophilic esophagitis [11]. A pediatric case series using an ultrasonography catheter probe demonstrated modest but significant increases in thickness of the combined mucosal-submucosa as well as muscularis propria in 11 children with EoE [31]. Straumann utilized endoscopic ultrasonography in a controlled trial of topical budesonide and demonstrated significant mural expansion in adults with EoE [32]. Doubling of the thickness of the mucosa and a 50% increase in the thickness of the muscularis propria were found with the most marked difference being a threefold increase in submucosal thickness. Consistent with the natural history of EoE being a chronic, progressive disease associated with submucosal remodeling, the magnitude of the relative increases in mural thickness demonstrated in adults is greater than found in children with EoE.

### Esophageal manometry

The expansion of the muscularis propria on EUS imaging as well as reports of dysphagia in EoE in the absence of identified esophageal stricture has led to the notion that esophageal motor function may be affected in EoE. Interestingly, the first two cases of “eosinophilic esophagitis” were reported in adults with major esophageal motility disorders; one having achalasia and the second having esophageal spasm [14]. These patients would be excluded from the current definition of EoE due to the presence of a major esophageal motility disorder and concomitant eosinophilic gastroenteritis but provide evidence for esophageal dysmotility in association with esophageal eosinophilia [33]. Additional reports of a muscular variant of EoE include a case in which esophageal resection specimen demonstrated marked esophageal mural expansion and eosinophilic infiltration with manometric findings of simultaneous esophageal body contractions with normal distal latency [11]. Another case report described an adult with more characteristic endoscopic and mucosal biopsy features of EoE with manometric findings consistent with achalasia that improved with systemic corticosteroids [34]. As noted above, a case series from Japan characterized five patients with hypercontractile esophageal dysmotility (Nutcracker or jackhammer) with eosinophilic inflammation of the muscularis propria identified on biopsies obtained during per oral esophageal myotomy [13]. Of note, these patients did not demonstrate typical features of EoE on endoscopy or mucosal biopsies. In contrast to these reports of increased contractility, other studies in adults with EoE have demonstrated both hypertensive and weak peristaltic function in a subset of EoE patients [35, 36].

An investigation of adults utilizing high-resolution esophageal manometry and Chicago classification systematically compared a cohort of 50 patients with EoE, 50 patients with GERD and 50 healthy controls and demonstrated normal peristalsis in 64%, with 36% demonstrating nonspecific esophageal motor patterns dominated by weak and failed peristalsis [37]. While such abnormalities could contribute to dysphagia, they are not accepted as major motility disorders due to generally poor correlation with symptoms. Furthermore, the frequency of these abnormal patterns was not significantly different from the motility abnormalities in the cohort of patients with GERD. A novel finding in this study was abnormal esophageal pressurization, characterized by pan esophageal pressurization in 16% and distal esophageal pressurization in 18%. Another study from Spain substantiated this observation through the demonstration of pan esophageal pressurization in 48% of EoE patients and none of a control group [38]. The esophageal pressurization events in EoE may reflect reduced esophageal mural compliance secondary to the transmural remodeling demonstrated on EUS imaging or alterations in motility that may occur secondary to EoE associated ESEA.

Esophageal motility evaluates esophageal circular muscle function but does not evaluate longitudinal muscle function that is responsible for axial movement of the esophagus. Using high-frequency ultrasonographic imaging, Korsapati and Mittal assessed longitudinal muscle activity in patients with EoE [39]. Compared with healthy controls, patients with EoE showed significantly reduced longitudinal muscle peak thickness as well as duration of contraction. These results are consistent with longitudinal muscle dysfunction in EoE. However, an alternate explanation of the defect identified is that the longitudinal muscle contractile activity is intact but that transmural remodeling alterations in EoE mechanically restrict the ability of the esophagus to shorten. Regardless of the underlying cause, impaired esophageal shortening can be a mechanism that limits effective esophageal bolus transport and thereby contributes to dysphagia and food impactions.

In summary, the available studies evaluating esophageal motor function in EoE have demonstrated both hypercontractile and hypocontractile esophageal body motor abnormalities that could impair esophageal bolus transport. The majority of manometric patterns identified are non-specific and do not meet criteria for accepted, major esophageal motility disorders. Jackhammer esophagus and esophageal spasm were specifically identified in patients with eosinophilic inflammation of the muscularis propria often without mucosal involvement. In most adults with EoE, dysphagia is likely the result decreased esophageal distensibility due to subepithelial inflammation and fibrosis. In addition to their possible clinical implications, manometric deficits in EoE provide important insights regarding the pathophysiologic effects of ESEA.

### Impedance planimetry

**Fig. 4:**
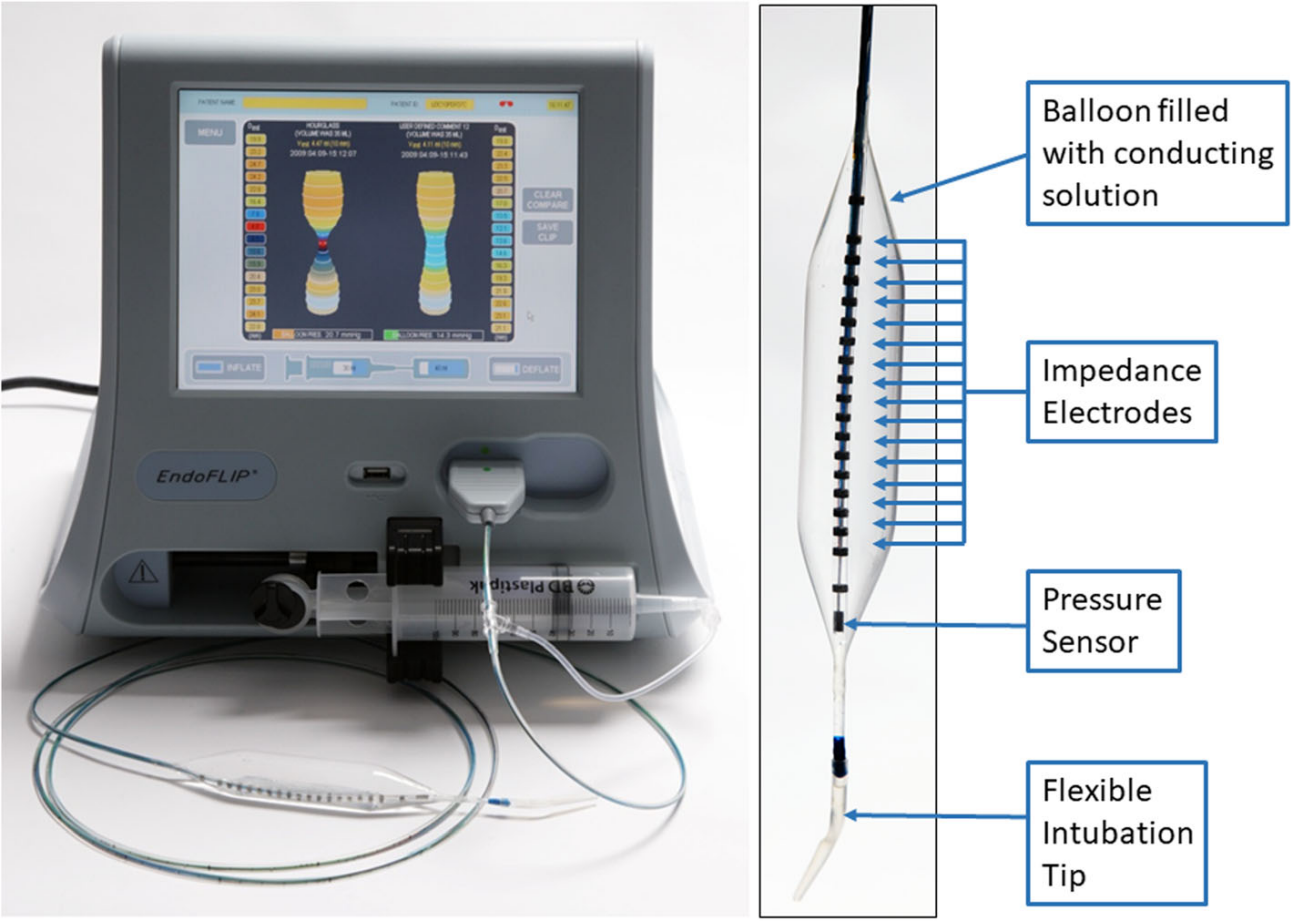
Impedance planimetry recording equipment (functional lumen imaging probe, FLIP). The left image shows the FLIP device which is a portable, bedside recording instrument with pump system to allow for volumetric distension of the gastrointestinal tract. The right image depicts the FLIP catheter that incorporates impedance electrodes that measure the cross-sectional area at 16 different longitudinally separated sites along with intraluminal pressure within an infinitely compliant balloon that fills with an electrode conducting solution

ESEA in EoE is inadequately assessed by endoscopy. Assessment of esophageal mural distensibility utilizing a impedance planimetry measured by functional luminal imaging probe (FLIP) is a better approach for quantifying the functional consequences of ESEA [40]. The FLIP technology incorporates a multichannel electrical impedance catheter and manometric sensor surrounded by an infinitely compliant bag that is filled with an electrode conducting solution (Fig. 4). As the bag is filled with the solution, the probe simultaneously measures the esophageal luminal diameter and pressure at multiple points along the catheter assembly. The resulting pressure–volume curves provide a detailed interrogation of the distensibility of the esophageal wall.

Recent investigations have applied FLIP to assess esophageal body biomechanics. Compliance plots were depicted as volume versus pressure while distensibility plots were depicted as cross-sectional area (CSA) versus intra-balloon pressure. Reduced distensibility at higher balloon pressures was observed leading to the metric of the distensibility plateau (DP) [41]. The DP, calculated by polynomial regression, represents the CSA of the esophagus at which increasing intra-luminal distension results in negligible increases in CSA. The calculation is based on the axial location within the recording region with the minimum CSA at a given distension volume. The narrowest CSA is a clinically relevant parameter as it defines the “rate limiting step” in esophageal bolus transit.

Esophageal distensibility characteristics in eosinophilic esophagitis (EoE) were first evaluated by Kwiatek et al. [41]. Distal esophageal body compliance was significantly reduced in EoE compared with controls. Interestingly, the compliance curves deviated during initial distension pressures under 20 mmHg but merged at higher pressures. Distensibility curves for the locus with the minimal CSA along an 8 cm segment of the distal esophagus demonstrated significant reductions in the DP in 33 patients with eosinophilic esophagitis compared with 15 controls. The overall DP was about 50% lower in EoE than controls (259 vs 438 mm^2^). Although variability in the DP was observed in both groups, 24 of 33(73%) EoE patients exhibited a DP of < 300 mm^2^, while 10 of 15 (67%) control subjects had a DP ≥ 400 mm2. Amongst patients with EoE, the DP was not significantly associated with the degree of esophageal eosinophilia. This latter observation is consistent with an important dissociation between esophageal mucosal eosinophilic inflammation and ESEA [42].

**Fig. 5:**
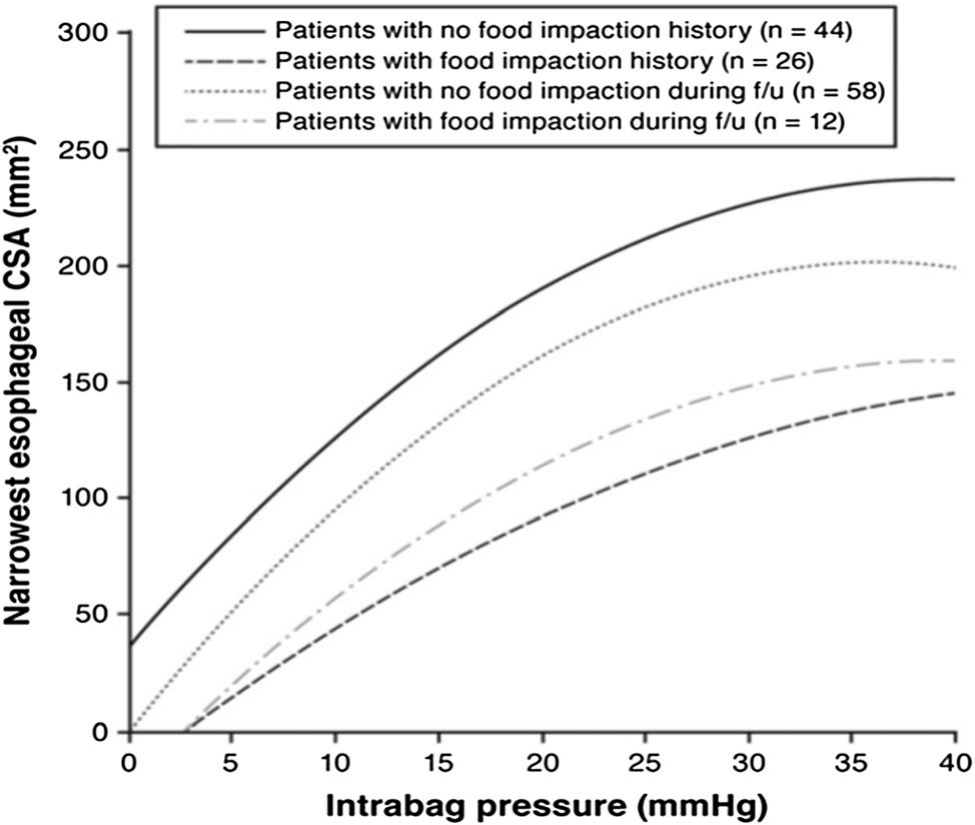
Impedance planimetry in eosinophilic esophagitis. Distensibility curves plot esophageal cross-sectional area along the most narrowed segment of the esophagus versus intraluminal distension pressure. Esophageal distensibility if reduced in EoE patients with food impaction compared to those without food impaction. This illustrates the relevance of esophageal subepithelial activity measured by impedance planimetry as related to clinical outcomes [3]

The clinical relevance of measurements of esophageal distensibility in EoE was demonstrated in a study utilizing impedance planimetry in 70 adults with EoE [3]. Esophageal distensibility curves as well as the DP were significantly lower in EoE patients with food impaction compared to EoE patients without food impaction. During a mean follow up period of 9 months, reduced esophageal distensibility was associated with the need for esophageal dilation and occurrence of food impaction (Fig. 5). A DP threshold of 225 mm^2^ (esophageal diameter of 17 mm) or less differentiated EoE subjects with food impaction. Esophageal eosinophilia was again not associated with food impaction outcomes or distensibility. The lack of correlation between esophageal distensibility and mucosal eosinophil density provides several potential insights into EoE disease mechanisms. Although epithelial eosinophilic inflammation is used to define one parameter of disease activity, it may not reflect the degree of submucosal activity. This is of relevance since the pathogenesis of remodeling lies largely below the mucosal surface. Mucosal eosinophilia is likely the harbinger of deeper tissue eosinophilia, as is seen in the LP and muscular layers in EoE. In turn, eosinophilia travels in conjunction with other inflammatory cells and mediators that drive fibrosis, angiogenesis, stenosis, and smooth muscle changes.

Application of impedance planimetry to children with EoE was recently reported and demonstrated reduced distensibility as well as association with food impaction [43]. Importantly, the analyses of control pediatric subjects demonstrated substantial variability in esophageal distensibility by age. This observation is logical given the expected increase in esophageal size as children grow. It also emphasizes the need for normative data to allow for accurate comparisons of disease activity across ages.

A comparison of EREFS and esophageal biomechanical properties on FLIP demonstrated that increased esophageal ring severity was significantly associated with reduced distensibility [30]. This observation implies that ring formation provides a visual estimate of the severity of underlying esophageal remodeling. On the other hand, the endoscopic features of exudate and furrows were associated with esophageal eosinophilia but not distensibility.

**Fig. 6:**
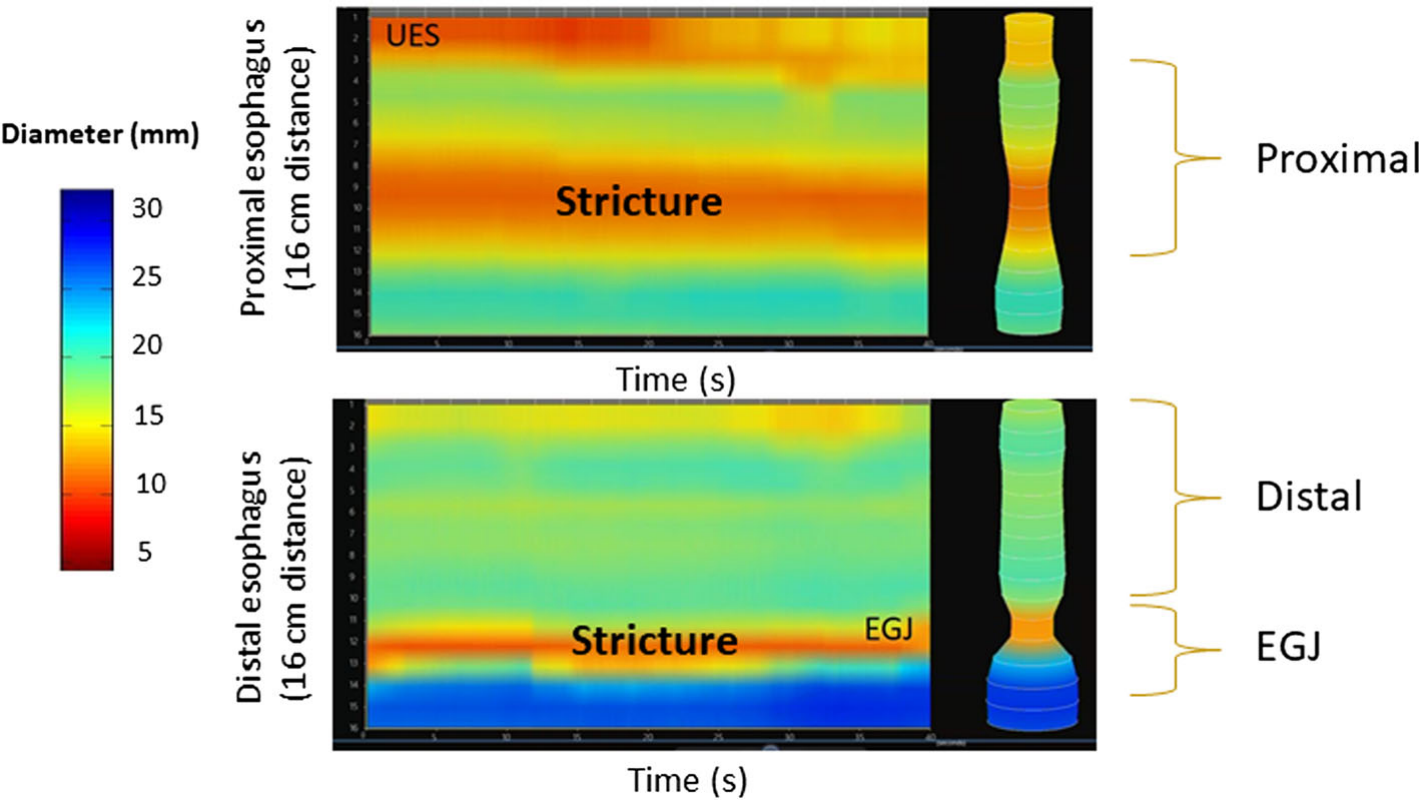
Impedance planimetry topographic plot mapping esophageal diameters over time with increasing volumetric esophageal distension. The upper plot depicts the upper 16 cm of the esophagus landmarked to the upper esophageal sphincter. The lower plot depicts the lower 16 cm of the esophagus landmarked to the lower esophageal sphincter. This plot demonstrates a focal stricture in the proximal esophagus with inner diameter of 10 mm in a patient with eosinophilic esophagitis

**Fig. 7:**
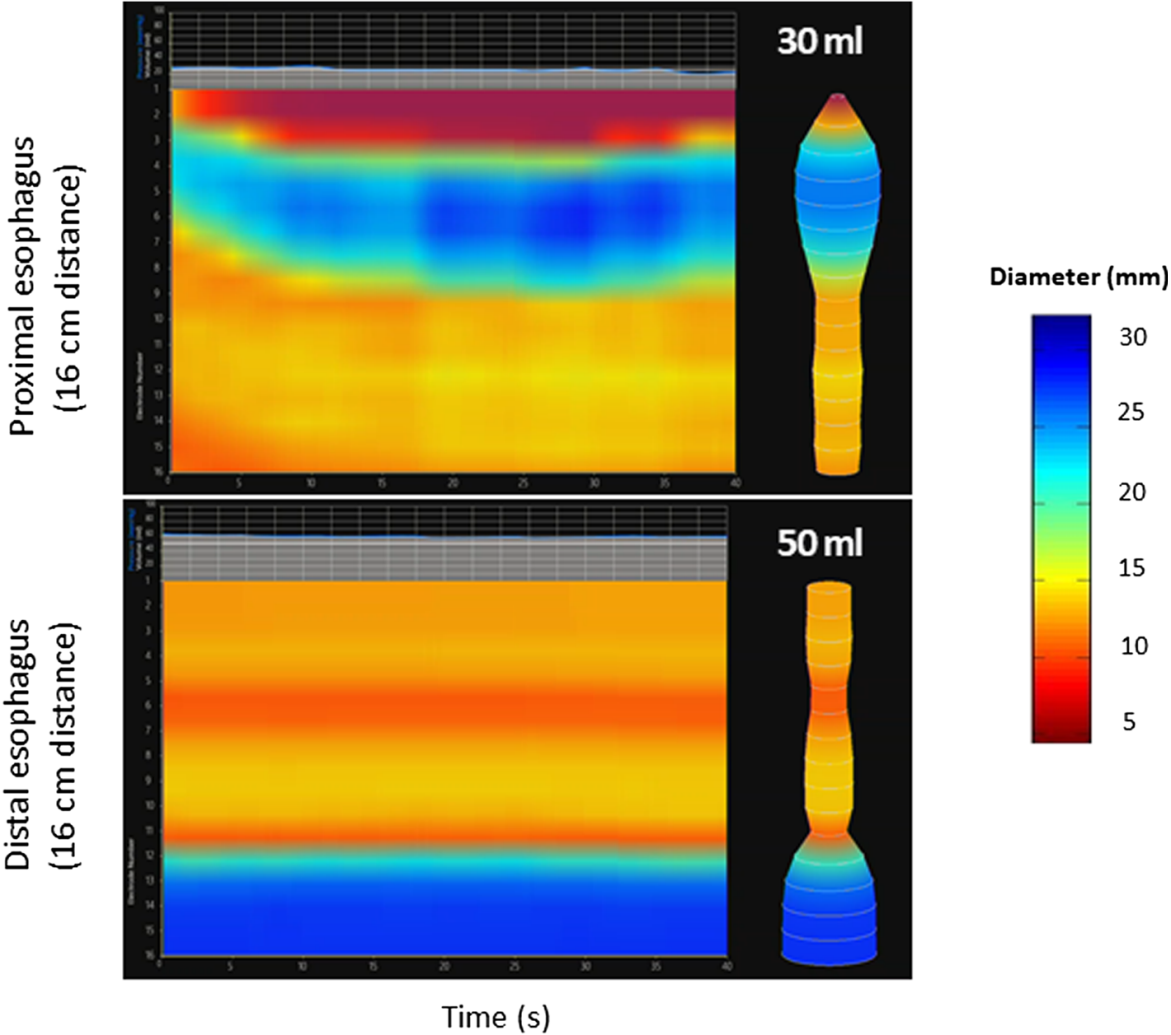
Impedance planimetry topographic plot illustrating diffuse narrow caliber esophagus in a patient with eosinophilic esophagus. With the exception of the upper 5 cm of the esophagus, the majority of the esophagus is less than 14 mm in diameter. The distensibility plateau in the distal esophagus is 10 mm

**Fig. 8:**
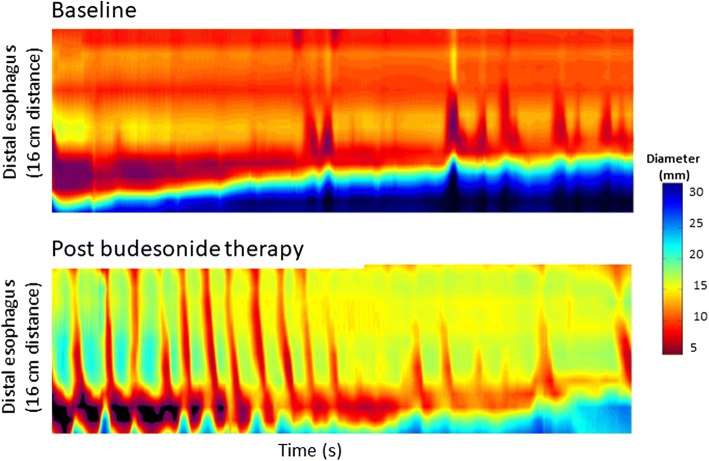
Impedance planimetry topographic plots of the distal esophagus in patient with eosinophilic esophagitis before and after swallowed topical budesonide administration. Prior to therapy, the patient demonstrates diffuse esophageal narrowing with a distensibility plateau of 7 mm and absent peristalsis. Following treatment, the distensibility plateau improved to 13 mm with more global improvement across the esophageal lumen. In addition, esophageal peristaltic function recovered [54]

The measurement of the DP is restricted to the narrowest CSA of the interrogated portion of the esophageal body. Esophageal distensibility topography captures a more comprehensive representation of the esophageal body [44]. Similar to high resolution esophageal manometric topography, the *y*-axis denotes the vertical distance along the esophagus whereas the *x*-axis depicts time during progressive, volumetric esophageal distension. Color topography illustrates the esophageal diameter. The pictorial representation allows for rapid recognition of patterns consistent with a focal esophageal stricture (Fig. 6) or diffuse esophageal luminal narrowing (“narrow caliber esophagus”) (Fig. 7). Improvement in esophageal distensibility following medical therapy is readily apparent on topography (Fig. 8).

In summary, the optimal clinical utilization of impedance planimetry measurements of esophageal body biomechanics in EoE is still being defined. The technology provides the most accurate and precise estimates for esophageal mural distensibility. Advantages over the barium esophagram include the ability to control esophageal distension pressure. On a practical note, impedance planimetry can be performed with acquisition times of less than 10 min during a routine endoscopy session, thereby obviating the need for scheduling a separate radiologic procedure. Most importantly, esophageal distensibility is significantly associated with the clinically relevant consequences of EoE, including strictures and food impaction. Thus, esophageal distensibility measurement may serve as an important biomarker of overall EoE activity in the future.

## Effectiveness of therapies for esophageal subepithelial activity in EoE

Therapeutic options in EoE include medications, elimination diets and esophageal dilation [45]. Pharmacologic therapies are commonly used in clinical practice but not yet approved by the United States Food and Drug Administration although several agents are in phase 3 clinical trials. A tablet formulation of budesonide was approved in 2018 by the European Medicines Agency. The primary endpoints used to judge the efficacy of therapies are symptoms and esophageal mucosal eosinophilic infiltration [46]. Symptoms are a consequence but indirect and often inaccurate measure of ESEA.

The benefits of swallowed topical steroids have been convincing in terms of resolving mucosal inflammation [45]. Available studies demonstrate heterogeneity regarding improvement in esophageal subepithelial fibrosis with swallowed topical steroids in EoE. Aceves first described significant reduction in the severity of fibrosis utilizing topical budesonide in children with EoE [47]. This observation was confirmed by two subsequent pediatric series that utilized diet and topical fluticasone as well as an adult study using topical budesonide [48-50]. Notably, the reduction in fibrosis paralleled improvement in epithelial inflammation. Improvement of fibrostenosis with steroids has been less consistent in adult studies of EoE. A significant improvement in esophageal fibrosis using a histopathologic fibrosis score was demonstrated in a randomized controlled trial of adults following 15 days of topical budesonide by Straumann [50]. Numerical but not significant improvement was reported in an uncontrolled, prospective study following a year of topical fluticasone [51]. While both Aceves (pediatric) and Straumann (adult) have shown reduction in TGFβ1 expression following topical steroid therapy, a study of fluticasone demonstrated decreases only in CCL18 [51]. The ability to improve fibrotic changes may depend on the degree and duration of fibrosis, the age of the patient, and formulation of topical corticosteroid.

Heterogeneity in histopathologic and biomarkers for subepithelial activity in EoE is also demonstrated in the clinical outcomes of controlled trials of topical steroids. Prospective studies in adults with EoE with both topical steroids have demonstrated symptom improvement but the persistence of endoscopically detected esophageal features of fibrostenosis including rings and strictures [50, 52, 53]. Swallowed budesonide significantly reduced esophageal subepithelial expansion quantified by endoscopic ultrasonography compared to placebo [32]. Furthermore, significant reduction in esophageal ring severity was demonstrated in a placebo-controlled trial of budesonide oral suspension [52]. Alexander and colleagues found improvements in esophageal lumen diameter in the subset of subjects with more restricted pre-treatment esophageal caliber following short-term topical budesonide [17]. A retrospective series of adults treated with medical or diet therapy for EoE demonstrated a significant improvement in esophageal distensibility, with 44% of patients achieving an increase in esophageal diameter by more than 2 mm (Fig. 8) [54]. The data, therefore, supports the ability for swallowed topical steroids to modestly improve ESEA in a subset of EoE patients.

Systemic therapy for EoE offers conceptual advantages over topical therapy with the potential to target subepithelial disease. To date, there have been three randomized studies in pediatric and adult EoE using anti-IL-5 and two trials targeting IL-13 and one trial targeting the IL-4 receptor. Decreases in epithelial TGFβ1 and tenascin C in adult subjects were demonstrated with anti-IL5 therapy [55-57]. Measurement of esophageal distensibility was an exploratory outcome of a recent phase 2 clinical trial of dupilumab targeting the IL-4 receptor. The trial demonstrated a significant increase in esophageal distensibility with dupilumab in addition to improvements in dysphagia, eosinophilic inflammation and endoscopic features [45].

Esophageal dilation is a highly effective means of alleviating symptoms of dysphagia in patients with EoE. Relief of dysphagia is immediate and associated with significant patient-reported satisfaction. Dilation, however, does not address the underlying inflammatory process responsible for the development of the stenosis [6]. Although relief of dysphagia can continue for over a year following esophageal dilation even in the absence of anti-inflammatory therapy, EoE is a chronic disease and symptomatic recurrence is expected in most patients [6]. Retrospective studies have shown that use of medical therapy to control eosinophilic inflammation was associated with reduced utilization of esophageal dilation in EoE [58].

## Conclusions

In conclusion, esophageal subepithelial activity (ESEA) in EoE is responsible for the major clinical symptoms and complications of EoE. As such, a reduction in ESEA may be an objective and clinically relevant endpoint of the therapeutics in EoE. The current emphasis on symptom outcomes inadequately recognizes the contribution of subepithelial disease and deemphasizes the importance of anti-inflammatory benefits in preventing future esophageal remodeling. Biomarkers and clinical techniques that provide information on subepithelial activity will more fully elucidate the potential benefits and limitations of therapeutic interventions. Currently available instruments for assessment of subepithelial activity in EoE are both limited and/or underutilized and thus represent an important, unmet clinical need.

## Abbreviations

EoE: Eosinophilic esophagitis
eos/hpf: Eosinophils/high-power field
DP: Distensibility plateau
GERD: Gastroesophageal reflux disease
PPI: Proton pump inhibitor
CSA: Cross-sectional area
FLIP: Functional lumen imaging probe
